# Nuclear Factor κB and Adenosine Receptors: Biochemical and Behavioral Profiling

**DOI:** 10.2174/157015911795596559

**Published:** 2011-06

**Authors:** Vickram Ramkumar, Krishna A Jhaveri, Xiaobin Xie, Sarvesh Jajoo, Linda A Toth

**Affiliations:** Department of Pharmacology Southern Illinois University School of Medicine P.O. Box 19629 Springfield, IL 62794, USA

**Keywords:** Adenosine, adenosine receptor, p50 knockout mice, NF-κB, sleep, caffeine.

## Abstract

Adenosine is produced primarily by the metabolism of ATP and mediates its physiological actions by interacting primarily with adenosine receptors (ARs) on the plasma membranes of different cell types in the body. Activation of these G protein-coupled receptors promotes activation of diverse cellular signaling pathways that define their tissue-specific functions. One of the major actions of adenosine is cytoprotection, mediated primarily via two ARs - A_1_ (A_1_AR) and A_3_ (A_3_AR). These ARs protect cells exposed to oxidative stress and are also regulated by oxidative stress. Stress-mediated regulation of ARs involves two prominent transcription factors - activator protein-1 (AP-1) and nuclear factor (NF)-κB – that mediate the induction of genes important in cell survival. Mice that are genetically deficient in the p50 subunit of NF-κB (i.e., p50 knock-out mice) exhibit altered expression of *A_1_AR* and *A_2A_AR* and demonstrate distinct behavioral phenotypes under normal conditions or after drug challenges. These effects suggest an important role for NF-κB in dictating the level of expression of ARs in vivo, in regulating the cellular responses to stress, and in modifying behavior.

## INTRODUCTION

Adenosine is an important inhibitory neuromodulator in the central nervous system (CNS). The major source of adenosine is ATP, a neurotransmitter or co-transmitter in the CNS; in the periphery, ATP is also released from mast cells, basophils and endothelial cells as a result of cellular damage. Other precursors of adenosine include ADP (released in large amounts by activated platelets) and cyclic AMP (a second messenger in most cells). Because the estimated ratio of ATP:AMP under normoxic condition is approximately 50:1, a small decrease in total ATP is expected to produce a large increase in concentrations of AMP and adenosine [1]. 

Adenine nucleotides are degraded by a series of ectonucleotidases. One such enzyme, 5’-nucleotidase, catalyzes the conversion of AMP to adenosine. This enzyme occurs both extracellularly (attached to the plasma membrane by glycosyl-phosphatidylinositol anchors) and in the cytosol [2]. In the CNS, it is located on glia and astrocytes near synaptic terminals. Adenosine is rapidly cleared from the extracellular space through a bi-directional facilitated transporter and/or through metabolism by adenosine deaminase to inosine. Inhibition of the activity of this transporter leads to increased extracellular adenosine and reduced excitatory synaptic transmission in rat hippocampal slices [3], affirming a CNS depressant action of the nucleoside.

AR subtypes include A_1_, A_2a_, A_2b_ and A_3_ [4]. The A_2A_AR is localized to the striatum, where it inhibits dopaminergic neurons [5]. Expression of the A_3_AR is low in rat CNS [6]; however, recent findings implicate A_3_AR in modulating CNS injury [7]. The A_1_AR is the predominant AR subtype in the CNS. Autoradiographical studies indicate wide distribution of the A_1_AR in CNS, particularly in cortex, hippocampus and thalamus [8]. The A_1_AR, is the primary mediator of the neuroprotective action of adenosine.

## 1. ADENOSINE AND ADENOSINE RECEPTORS AND CYTOPROTECTION

Adenosine is released in response to ischemic stress and activates cells in the vicinity of its release site, thus serving a paracrine role. Extracellular concentrations of adenosine correlate with cerebral blood flow. For example, a transient increase in adenosine occurs when flow falls below 25 ml 100g^–1^ min^–1^ [9]. Increased adenosine confers protection against transient cerebral ischemia. For example, administration of 2-chloroadenosine protected rats from ischemia-induced hippocampal cell loss [10]. Another adenosine analog, cyclohexyladenosine, protected against cerebral ischemia in gerbils [11] and transient ischemia in rats [12]. Cyclohexyladenosine also provided protection to the hippocampus and striatum after 30 min of bilateral carotid occlusion [12]. Rats treated with caffeine, which increases A_1_AR expression in the brain, were more resistant to ischemia, underscoring the protective role of this receptor subtype [13]. In contrast, prolonged agonist treatment reduced A_1_AR expression and exacerbated the damage created by a subsequent ischemic episode [14]. Overall, these studies implicate the A_1_AR in neuroprotection under ischemic conditions.

Several mechanisms have been proposed to contribute to the cytoprotective role of adenosine. The major mechanism involves activation of presynaptic A_1_AR to decrease release of excitatory neurotransmitters such as glutamate [15-17]. These presynaptic A_1_AR activate K^+^ conductance, leading to hyperpolarization [18, 19] and inhibition of Ca^2+^ influx into the nerve terminal [12]. Such actions reduce neuronal excitability and firing rate [20]. Adenosine-mediated activation of a voltage-dependent Cl^-^ conductance that is distinct from that activated by GABA can also contribute to neuronal hyperpolarization [21]. Adenosine also acts postsynaptically to reduce NMDA receptor-induced synaptic amplification and hyperpolarizes astrocytes [22], thereby facilitating glutamate uptake by these cells. In addition, adenosine acts as a vasodilator in most vascular beds and augments cerebral blood flow [23]. A more delayed protective action of the A_1_AR is evident in its role in scar formation and subsequent tissue remodeling [24].

In contrast to the A_1_AR, activation of the A_2A_AR exacerbates neuronal damage, as inferred from various observations of a protective role of A_2A_AR blockade in different models of ischemia [25-29]. In addition, genetic deletion of the A_2A_AR protected against transient focal brain ischemia in mice [30]. The proposed mechanism(s) underlying the protective action of A_2A_AR blockade could be broadly classified as inhibition of glutamate toxicity and reduction in inflammation. The primary action of A_2A_AR activation is suggested to be a reduction of glutamate transport into [31] or its release from astrocytes [32]. The A_2A_AR has also been implicated in the recruitment and activation of microglia in the brain in response to injury or stress (for review, see 29), supporting therapeutic potential for receptor blockade by antagonists.

Studies focusing on the A_3_AR in the brain indicate that its activation can cause either protection from or exacerbation of neuronal injury. For example, activation of the A_3_AR reduces ischemic preconditioning and promotes synaptic failure produced by oxygen and glucose deprivation in the rat hippocampus [7]. This apparent toxicity of A_3_AR agonists was associated with rapid receptor desensitization after the ischemic episode. In contrast, A_3_AR agonists were protective after a shorter period of ischemia (2-5 min) in the absence of significant A_3_AR desensitization [33]. However, activation of the A_3_AR reduced ischemic brain damage after transient ligation of the middle cerebral artery [34]. The A_3_AR has also been implicated in protecting cultured astrocytes from hypoxic damage [35]. The basis of these differing roles of the A_3_AR is not clear, but could be related to the degree of receptor activation [36]. Accordingly, a lower level of A_3_AR activation may confer protection, whereas a higher level of activation produces cytotoxicity [36].

## 2. ADENOSINE RECEPTOR AND OXIDATIVE STRESS

Over the past several years, our laboratory has studied mechanism(s) underlying the cytoprotective effects of adenosine. We have shown that adenosine contributes to cytoprotection by enhancing the activities of antioxidant enzymes (e.g., superoxide dismutase, catalase and glutathione peroxidase) *via* activation of their protein kinase C-mediated phosphorylation [37]. This mechanism could reduce the level of reactive oxygen species (ROS), which could be harmful to the cell, appears to be relevant *in vivo*, and may account for adenosine-induced reduction of lipid peroxidation in the cochlea [38]. The nonselective adenosine analog (*R*-phenylisopropyladenosine) protects cochlear explants from damage induced by cisplatin [39] and chinchilla cochlea from noise-induced loss of hair cells, presumably *via* the A_1_AR [40]. Activation of the A_1_AR also protects against cisplatin-induced toxicity [41] and noise-induced hearing loss [42].

The A_1_AR is responsive to oxidative stress. Not only does adenosine suppress ROS generation, but ROS induce *A_1_AR* expression. This feedback induction of the *A_1_AR* boosts the A_1_AR response during high oxidative stress and is mediated by the stress-regulated transcription factor, nuclear factor-κB [43]. NF-κB-dependent transcription is a novel mechanism by which cellular oxidative stress modulates A_1_AR and G protein-coupled receptors. This mechanism could influence drug responses in cells under oxidative stress. In addition, NF-κB has similar influences on *A_2A_AR* expression [44]. Details of these findings and their behavioral manifestations in mice in response to drug challenges are discussed below.

## 3. NUCLEAR FACTOR κB FUNCTION AND REGULATION

The transcription factor NF-κB, first described for its role in the transcription of the immunoglobulin κ light chain in B lymphocytes [45], mediates gene expression in response to a variety of stimuli. The mammalian NF-κB family consists of five members, p65 (RelA), RelB, c-Rel, p50/p105 (NF-κB1) and p52/p100 (NF-κB2), all of which possess a common Rel homology domain [46]. The Rel homology domain is a conserved region of 300 amino acids present at the N-terminal of these proteins and serves multiple functions, including DNA binding, dimerization, and interaction with the inhibitory subunit, IκB. The Rel domain also contains the nuclear localization sequence that allows nuclear translocation of the subunits after NF-κB activation [47]. In the inactive state, NF-κB exists in the cytoplasm as a homo- or heterodimer bound to IκB. The associated IκB can become phosphorylated secondary to stimuli that activate IκB kinase (IKK). Phosphorylation of IκB results in its ubiquitination and degradation, which releases the dimer for nuclear translocation and stimulation of the transcription of genes that contain the decameric κB consensus sequence. 

The p65/p50 combination is the most abundant NF-κB dimer in most cells. Other transcriptionally active dimers are p65/p65, p50/c-Rel and p65/c-Rel. Some dimer combinations, such as p50/p50 and p52/p52 are believed to be inactive or repressive [48]. However, these dimers can stimulate transcription by binding to an IκB-like nuclear protein, BCL-3 [49]. The p50 subunit lacks the transcriptional activation domain that is present in p65 and c-Rel subunits, which likely accounts for property of transcriptional repression by the p50 homodimer [50].

In addition to IκB phosphorylation, dissociation, and degradation, other mechanisms can activate NF-κB. The p-100 mediated pathway involves an NF-κB inducing kinase (NIK) and IKK1. IKK1 phosphorylates p100, which leads to its ubiquitination and degradation to form the p52 subunit. The p52 subunit can then dimerize with RelB, and the dimer can enter the nucleus. In a similar fashion, constitutive processing of p105 to produce p50 occurs in the cytoplasm, allowing formation of p50/p50 dimers, which can then enter the nucleus [51].

## 4. NUCLEAR FACTOR κB REGULATION OF A_1_AR AND A_2A_AR EXPRESSION

### 4.1. Adenosine A_1_ Receptor

Early studies in our laboratories indicated that the A_1_AR is dynamically regulated by oxidative stress. In effect, the A_1_AR serves as a sensor of oxidative stress. These observations were initially derived from studying the effect of the chemotherapeutic agent cisplatin on the expression of A_1_AR in the chinchilla cochlea [38]. Cisplatin damages outer hair cells of the cochlea and produces significant hearing loss. We observed significant induction of A_1_AR in the cochlea prior to the induction of outer hair cell death. This induction likely represents a mechanism for combating, albeit ineffectively, the toxic effect of cisplatin in the cochlea.

To delineate the mechanism responsible for *A_1_AR* induction, we examined the role of NF-κB, which is known to be induced by oxidative stress. Using the inhibitor pyrrolidine dithiocarbamate (PDTC), we showed that cisplatin-induced induction of A_1_AR was directly related to activation of NF-κB. Induction of the *A_1_AR* also occurred in response to other chemotherapeutic agents (e.g., doxorubicin, mitroxantrone) that increase the production of ROS. Cisplatin-induced A_1_AR induction in cells was blunted by incubation with the ROS scavenger catalase or the inhibition of NF-κB with dexamethasone. Examination of the promoter of the human *A_1_AR *gene suggested the presence of an NF-κB consensus sequence located 623 base pairs upstream of the start site of promoter A construct (pBLPnif/PmtA) [52]. Transfection of a plasmid construct containing this promoter and firefly luciferase reporter gene in ductus deferens tumor (DDT) cells allowed us to demonstrate NF-κB dependent regulation of promoter activity [43]. These data suggest that oxidative stress, through activation of NF-κB, could induce the expression of the *A_1_AR*. Such a process would likely aid in cytoprotection under conditions of increased oxidative stress.

A_1_AR are also induced in response to other conditions associated with oxidative stress and inflammation. For example, A_1_AR mRNA and protein increased in brain after the induction of cerebral ischemia in rats [53]. In addition, nitric oxide acts through NF-κB to increase A_1_AR expression in a pheochromocytoma cell line (PC12 cells) and in isolated cortical neurons [54]. Noise exposure, which is associated with increased NADPH oxidase activity in the chinchilla cochlea, is also linked to the induction of the A_1_AR in the inner ear [55]. A_1_AR and A_3_AR were also induced in the gut in a rabbit model of ileitis [56], and A_1_AR was induced in kidney in association with oxidative stress produced by osmotic diuresis [57].

### 4.2. Adenosine A_2A_ Receptor

Gene expression profiling in PC12 cells demonstrated that exposure to nerve growth factor (NGF) significantly reduced the expression of the *A_2A_AR* gene. The initial study reported a 3-fold decrease in A_2A_AR within 3 days of NGF treatment [58]. Further studies demonstrated that NGF caused rapid activation of NF-κB *via* the low affinity p75 NGF receptor, with a resultant reduction in A_2A_AR [44]. This response was mimicked by agents that activate NF-κB (e.g., ceramide, H_2_O_2_) [44]. These results support a functional role for the NF-κB consensus sites present in the *A_2A_AR* gene promoter [59]. Other mechanisms for NGF regulation of A_2A_AR depend on TrkA-, Src-, and Ras and are linked to the activation of extracellular regulated kinase and stress-activated protein kinase/c-Jun NH(2)-terminal kinase [60]. The involvement of multiple pathways in the regulation of the A_2A_AR by NGF may indicate different temporal profiles of activation of these pathways during development. In addition, the p75/NF-κB pathway could provide a general mechanism for control of *A_2A_AR* expression by neurotrophins that do not use TrkA for signaling (e.g., brain-derived neurotrophic factor).

## 5. A_1_AR EXPRESSION AND FUNCTION IN P50 KNOCKOUT MICE

To evaluate the significance of NF-κB in the regulation of the *A_1_AR* expression, we used mice with deletion of the gene for the p50 subunit of NF-κB. Although this gene knock-out (KO) renders these mice immune deficient, they are viable and able to reproduce [61]. However, deletion of the p65 subunit of NF-κB is embryonically lethal. Deletions of p65 can therefore generally be assessed only *in vitro* [62].

### 5.1. Biochemical Characterization

**Fig. (1).**
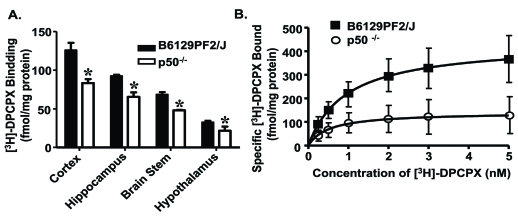
**A_1_AR in brain of p50 KO mice.** (**A**) A1AR binding in F2 and p50 KO mice (*n* = 4 per strain) was quantified in membrane fractions of cortex, hippocampus, brain stem, and hypothalamus using the specific A_1_AR antagonist [^3^H]-DPCPX (1 nM). Values are expressed as fmol/mg protein and represent mean ± S.E.M. for three independent experiments with samples assayed in triplicate (* *p* < 0.05, Student’s *t*-test). (**B**) Saturation binding analysis of cortical membrane for A_1_AR with [^3^H]-DPCPX in the absence (total binding) or presence (nonspecific binding) of 0.5 mM theophylline (n = 5 per strain). Curves were fitted to a one-site model using GraphPad Prism. Modified from Jhaveri *et al.*, **2007**, *Neuroscience 146*, 415-426, with permission from Elsevier.

Brain cortical plasma membranes prepared from p50 KO mice demonstrated significant reductions in the levels of the A_1_AR, as determined by radioligand binding assays, Western blotting, immunohistochemistry, and real time polymerase chain reactions (PCR) [63] (Fig. **1**). Similar reductions in A_1_AR were measured in the hippocampus, brain stem, hypothalamus and peripheral tissues (adrenal gland, kidney and spleen) Fig. (**1**). Levels of the guanine nucleotide coupling proteins (G proteins) alpha subunits (Gα_i3_) were also significantly reduced in the p50 KO mice, although the levels of Gα_i1_ were not changed, suggesting deficits in multiple components of the A_1_AR signaling cascade. Functionally, the deficit in A_1_AR/G_i_ protein expression was associated with reduced protection of neurons from apoptosis [63]. Overall, these findings suggest that activation of NF-κB is essential for maintaining normal *A_1_AR* expression and function and for survival of neurons in culture. Thus, A_1_AR promotes neuronal survival.

### 5.2. Sleep

Adenosine and AR have been extensively studied for their involvement in the regulation of sleep and wakefulness. Previous studies established adenosine as a sleep-inducing factor that increases in the basal forebrain during prolonged wakefulness [64]. Similarly, reduced expression of *A_1_AR* in the magnocellular cholinergic region of the basal forebrain by antisense oligonucleotides alters sleep [65]. However, recent studies indicate a complex role for A_1_AR in mediating sleep and arousal. For example, evaluation of A_1_AR and A_2A_AR KO mice showed that caffeine promotes arousal through its action at the A_2A_AR, but not the A_1_AR [66]. However, a subsequent study by the same group found that activation of the A_1_AR in the tuberomammillary nucleus could mediate non-rapid eye movement (non-REM) sleep through suppression of histamine release [67].

**Fig. (2).**
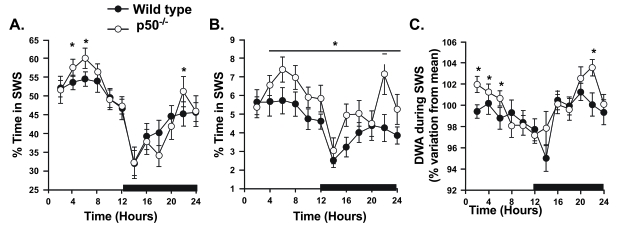
**Patterns of sleep in B6129PF2/J (wild type) and p50 knockout (KO) mice.** The amount of time spent in slow-wave sleep (SWS; **A**) and rapid-eye-movement sleep (REMS; **B**) are expressed as percentage of recording time spent in each state. Delta wave amplitude during SWS (**C**) is expressed as a percentage of mean values measured over the entire 24 h recording period. The black bar along the x-axis denotes the dark phase of the diurnal cycle. Solid circles, F2 (wild-type) mice (*n* = 16); open circles, p50 KO mice (*n* =14). Each datum point denotes a 2-h average ± S.E.M. *Significance at the specified time point (*p* < 0.05 by ANOVA and independent Student’s *t*-test). Modified from Jhaveri *et al.* (2006), *Am J Physiol Regul Integr Comp Physiol 291*, R1516-1526, with permission from the American Physiological Society.

We evaluated p50 KO mice to determine whether their deficit in the A_1_AR would affect sleep (Fig. **2**). The p50 KO mice had more slow wave and rapid eye movement (REM) sleep under normal conditions than did wild type mice (B6129PF2/J strain) [68]. This finding was surprising, because based on the purported role of the A_1_AR in regulating sleep in rats [65], less *A_1_AR* expression should be associated with reduced sleep duration. Dysregulation of another AR (such as the A_2A_AR) or other factors (such as increased inflammatory cytokine or prostaglandin D_2_ production) in the p50 KO mice could account for this finding. In addition, p50 KO mice showed an accelerated return to normal sleep after sleep deprivation. Finally, p50 KO mice showed a greater suppression of SWS after administration of bacterial lipopolysaccharide (LPS) challenge. These data support a role of NF-κB in regulating normal sleep and mediating the responses to sleep deprivation and immune challenge. However, the involvement of AR in mediating the sleep profiles of p50 KO mice remains undetermined. 

In addition to differences in sleep, the p50 KO mice generally exhibited lower core body temperatures than did the wild-type mice. LPS produced a transient hyperthermia in wild type mice, but persistent hypothermia in p50 KO mice [68]. This finding suggests that the p50 KO mice either lacked a normal homeostatic response to hypothermia or developed a more prolonged and severe response to LPS, in either case implicating NF-κB in the normal reaction. This finding is also interesting in light of a reported temperature-dependent activation of NF-κB in mouse liver [69]. LPS-stimulated TNF-( levels were higher in p50 KO versus the wild type mice, which could also contribute to the persistent hypothermia observed in the p50 KO mice. 

## 6. A_2A_AR EXPRESSION AND FUNCTION IN P50^-/-^ MICE

### 6.1. Biochemical Characterization

**Fig. (3).**
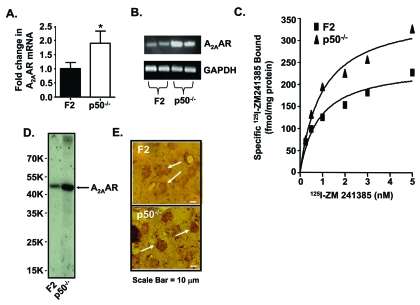
**A_2A_AR in striata of p50 KO versus F2 mice.** (**A**) Real time PCR (A; n = 10 per group). (**B**) Traditional PCR (n = 2 per group). (**C**) Saturation curves with inset. Scatchard plot for ^125^I-ZM241385 binding to A_2A_AR in mouse crude striatal membranes. The plots shown represent pooled striata from 4 mice, with samples at each concentration assayed in triplicate. This analysis was repeated on 3 different pooled samples with similar results. (**D**) Affinity purification of the A2AAR from F2 and p50 KO striata (n = 4 per strain). **E**) Immunohistochemistry of striatal A_2A_AR from F2 and p50 KO mice (n=4 per strain). Arrowheads depict specific A_2A_AR-immunolabeled neurons. The sections represent a 400-fold magnification of the image. Scale bar represents 10 µm. Reprinted from Xie *et al.*, **2007**, *Life Sci 81*, 1031-1041, with permission from Elsevier.

A_2A_AR expression in brain is localized largely in the striatum. Striatal A_2A_AR, together with dopamine D_2_ receptor (D_2_R), participates in the control of locomotor activity. These receptors demonstrate mutual antagonistic actions by physically interacting with each other and differentially modulating post-receptor signal transduction pathways. As such, inhibition of the A_2A_AR by caffeine promotes hyperactivity by reducing adenosine inhibition of D_2_R function. Recent *in vitro* evidence indicates that expression of the *A_2A_AR* [44] and the *D_2_R* [70] are differentially regulated by NF-κB, highlighting another potential difference between these two receptors. To extend these observations, we evaluated the *in vivo* regulation of these receptors using p50 KO mice, with the wild type B6129PF2/J (F2) mice serving as controls (Fig. **3**). Quantification of adenosine receptor (AR) subtypes in striata from p50 KO mice using PCR, radioligand binding assays and immunocytochemistry showed more A_2A_AR mRNA and protein, but less A_1_AR mRNA and protein as compared with F2 mice. Striata from p50 KO mice also showed less D_2_R mRNA and [^3^H]-methylspiperone binding [71]. These studies suggest that absence of the NF-κB p50 subunit leads to dysregulation of ARs and D_2_R in the striatum, as observed for the A_1_AR in other brain regions. Overall, these studies suggest a role of the NF-κB p50 subunit and/or the composition of the heterodimers in regulation of striatal A_1_AR, A_2A_AR and D_2_R.

### 6.2. Caffeine-Induced Locomotor Activation

**Fig. (4).**
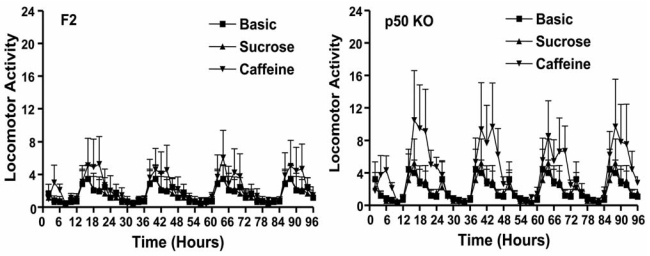
**Locomotor activity of p50 KO and F2 mice in response to caffeine ingestion.** Mice of both strains were randomly assigned into caffeine treatment or control groups (n=14 per group). Caffeine was administered in drinking water (300 mg/L) in combination with sucrose (20 g/L). Control groups received only sucrose (20 g/L) in drinking water. Mice received sequentially regular drinking water for 48 h (black circles), sucrose only in drinking water for the next 48 h (red circles), and caffeine with sucrose for the final 96 h (blue circles). Reprinted from Xie *et al.*, 2009, *Life Sci 85*, 226-234, with permission from Elsevier.

Alterations in the balance of A_2A_AR and D_2_R function in striatum could alter locomotor activity. Although F2 and p50 KO mice did not differ in terms of basal locomotor activity, the p50 KO mice demonstrated hypersensitivity to caffeine-induced behavioral activation during the dark phase (when mice are normally active) but not during the light phase (when mice are normally asleep) (Fig. **4**) [72]. Similarly, p50 KO mice showed behavioral hypersensitivity to intraperitoneal injections of SCH58261, an A_2A_AR antagonist [72]. In contrast, intraperitoneal injections of the selective A_1_AR antagonist, DPCPX, increased locomotor activity in both F2 and p50 KO mice, indicating that the increased sensitivity of p50 KO mice to caffeine likely did not involve the A_1_AR. The intraperitoneal administration of a combination of SCH 58261 and DPCPX produced an apparent synergistic increase in locomotor activity in both strains of mice. Administration of the D_2_R antagonist, raclopride, reduced the stimulatory effect of SCH58261 on locomotor activity. This latter finding suggests an involvement of D_2_R activation in mediating locomotor activation after A_2A_AR blockade. The locomotor activity data support several conclusions: 1) the p50 KO mice are more sensitive to A_2A_AR antagonists than are wild type F2 mice, 2) the higher striatal A_2A_AR in KO mice could provide a larger striatal target for inhibition by caffeine, thus accounting for the heightened behavioral sensitivity and 3) the increased sensitivity to A_2A_AR antagonists results, at least in part, from increased D_2_R activity.

## 7. CONCLUSION

**Fig. (5).**
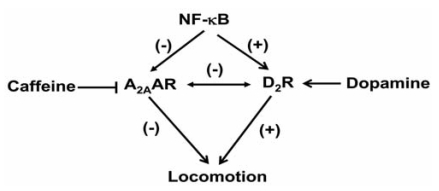
**Proposed model of NF-κB interactions with different A_2A_AR and D_2_R in the striatum.** Activation of NF-κB decreases A_2A_AR expression but up regulates D_2_R expression and thereby influences the contribution of these receptors to locomotion. Such a model could contribute to differing efficacies of drugs such as caffeine (an antagonist of the A_2A_AR) and D_2_R agonists (such as dopamine).

NF-κB plays a significant role in the regulation of genes involved in response to immune activation and oxidative stress. To date, only a few studies have detailed the involvement of this transcription factor in regulation of G protein coupled receptors. In this review, we describe differential regulation of the *A_1_AR* and *A_2A_AR* by NF-κB and show that these receptors are differentially expressed in mice deficient in the p50 subunit of NF-κB. Behavioral studies performed on these mice suggest a role for the p50 subunit and the A_1_AR in the regulation of normal sleep, the response to sleep deprivation, and the sleep patterns that develop after an immune challenge. The p50 KO mice also show behavioral sensitivity to caffeine and A_2A_AR antagonists, which may be related to the greater expression of the *A_2A_AR* in the striatum and possibly to increased dopamine release and D_2_R activity in the KO mice (Fig. **5**). However, the potential for various A_1_AR, A_2A_AR and D_2_R heterodimer combinations [73] with different functions in the striatum of the p50 KO mice complicates interpretation of their behavioral hypersensitivity to caffeine. Nevertheless, the p50 KO mice could provide a useful model for studying the expression and integration of multiple NF-κB targets in the regulation of behavior.
